# Awareness of Diabetic Ketoacidosis and Adherence to Diabetes Self-Care Practices Among Patients With Type 1 Diabetes Mellitus at King Fahad Specialist Hospital in Buraidah, Saudi Arabia

**DOI:** 10.7759/cureus.112939

**Published:** 2026-07-19

**Authors:** Nagwa A Fattouta, Omar K Alsaawi, Abdullah K Alsaawi, Ali F Almetrafe, Yazeed I Almershed, Abdulaziz H Alburaidi, Muath Aldukhayel

**Affiliations:** 1 Diabetes and Endocrinology, Alrass General Hospital, Alrass, SAU; 2 Internal Medicine, King Fahad Specialist Hospital, Buraidah, SAU; 3 Family Medicine, Third Health Cluster, Saudi Ministry of Health, Riyadh, SAU; 4 Family Medicine, Qassim University Medical City, Buraidah, SAU

**Keywords:** adherence, diabetes-related complications, diabetic ketoacidosis, dka awareness, patient education, saudi arabia, symptom recognition, type 1 diabetes

## Abstract

Background and objective

Diabetic ketoacidosis (DKA) remains a major acute complication in patients with type 1 diabetes. Despite advances in diabetes management, recurrent DKA remains an issue and is often driven by knowledge gaps in symptom recognition, management awareness, and adherence to recommended self-care practices. We aimed to assess awareness of DKA symptoms, management, and diabetes-related complications, as well as adherence to recommended care practices among patients with type 1 diabetes attending outpatient clinics at King Fahad Specialist Hospital (KFSH) in Buraidah, Saudi Arabia.

Methodology

This cross-sectional study included 206 patients with type 1 diabetes. Data were collected through a structured questionnaire that evaluated participants' awareness of DKA symptoms, its management and prevention, diabetes-related complications, and adherence to recommended diabetes self-care practices. Descriptive statistics, correlation analyses, and comparative analyses across different sociodemographic groups were conducted.

Results

Of the 206 patients, 104 (50.5%) were aged 21-25 years, and 114 (55.3%) were men. Awareness of DKA symptoms was moderate in 130 (63.1%) patients, and awareness of DKA management and prevention was also predominantly moderate in 148 (71.8%) patients, whereas awareness of diabetes-related complications was generally poor, with only 46 (22.3%) patients demonstrating moderate knowledge. Adherence was moderate among 112 (54.4%) patients, with stronger adherence to clinical follow-up practices than to lifestyle-related behaviors. Significant positive correlations were observed between all awareness domains and adherence (p < 0.01). Women had significantly higher awareness of DKA symptoms (p = 0.046) and significantly higher adherence to diabetes care (p < 0.001). Insulin pump users demonstrated markedly higher awareness across all domains (p < 0.001) and higher adherence (p = 0.021). Patients with HbA1c ≤ 8% also showed significantly higher awareness of DKA symptoms and management (p < 0.05) and substantially better adherence (p < 0.001). Awareness of diabetes complications was significantly higher among older patients (aged ≥ 26 years; p = 0.009) and those with higher education (p = 0.018).

Conclusions

Important gaps in patient awareness of severe DKA symptoms, secondary precipitation factors, and diabetes-related complications were observed. Clinical monitoring practices were generally adequate, but lifestyle adherence was limited. Women, insulin pump users, and patients with optimal glycemic control (HbA1c ≤ 8%) consistently demonstrated higher awareness and adherence, suggesting an association with better diabetes self-management. The strong association between awareness and adherence highlights the need for structured, repeated, and culturally sensitive diabetes education programs. Increasing patient and caregiver knowledge may improve diabetes self-management and may potentially contribute to reducing DKA recurrence and improving long-term outcomes.

## Introduction

Diabetic ketoacidosis (DKA) is a serious, yet preventable, acute complication of diabetes mellitus, accounting for a significant proportion of emergency admissions and prolonged hospitalizations worldwide. DKA is characterized by an absolute or relative deficiency of insulin, resulting in hyperglycemia, ketonemia, and metabolic acidosis. Common precipitating factors include infection, insulin omission, and new-onset diabetes [1]. International clinical guidelines emphasize that early recognition of DKA symptoms, including polyuria, polydipsia, abdominal pain, vomiting, and altered mental status, along with prompt initiation of treatment, can substantially reduce morbidity and improve clinical outcomes [2,3]. Despite these well-established recommendations, DKA occurs frequently, particularly in patients with poor glycemic control or a limited understanding of self-management principles.

Inadequate awareness and poor adherence to recommended self-care practices are major contributors to preventable DKA episodes. Behavioral and psychosocial factors, including insulin nonadherence, poor health literacy, and limited engagement in diabetes education programs, are strongly associated with recurrent DKA in international cohorts [4]. Similar findings have been reported regarding diabetes self-care. Structured education, empowerment, and continuous counseling have been shown to improve glycemic control and reduce acute complications, underscoring the critical role of patient knowledge in preventing DKA [5]. More recent multicenter European data indicate that knowledge of DKA symptoms, precipitating factors, and sick-day rules is suboptimal, even among individuals with type 1 diabetes, with significant gaps in the recognition of early warning signs and self-management [6]. These findings suggest that awareness and adherence are not independent constructs but rather interdependent determinants of DKA risk.

Saudi Arabian studies provide an important local context and indicate persistent deficits in DKA-specific knowledge among patients and their caregivers. One Riyadh study reported that most patients with diabetes had poor knowledge of DKA symptoms, triggers, and prevention strategies, with younger adults having particularly poor levels of understanding [7]. Limited knowledge of DKA was also observed among patients with diabetes and their caregivers in a large survey conducted in Makkah, highlighting the need for structured educational interventions [8]. Studies involving caregivers in Khobar and other regions also reported that the parents of children with type 1 diabetes frequently lacked important knowledge regarding the recognition of early symptoms and the appropriate actions to take in the event of acute illness, thus increasing their children's risk of severe DKA [9,10].

Low awareness of DKA, including knowledge and preventive practices, has been reported in large surveys conducted in Saudi Arabia’s northern and western regions, supporting the notion that educational gaps are common across demographic groups and geographic areas [11]. Moreover, a Saudi national survey reported that a large proportion of patients had poor knowledge of DKA, further confirming that these educational gaps were not limited to a specific region or facility [12]. These findings indicate a substantial discrepancy between recommended practices and actual awareness in Saudi Arabia, despite international guidelines recommending routine sick-day education, early recognition of symptoms, and preventive counseling [2,3].

Poor awareness and nonadherence globally, as predictors of recurrent DKA [4], may be related in Saudi Arabia to higher rates of preventable complications attributable to consistently low awareness across Saudi cohorts. Given this background, healthcare institutions with large diabetic populations need to evaluate DKA awareness levels and their association with adherence. King Fahad Specialist Hospital (KFSH) in Buraidah serves a diverse patient population in the Qassim region. Limited local data are available regarding awareness of DKA, knowledge of its prevention, and the correlation between awareness and adherence. Understanding these associations is critical for designing targeted educational interventions, improving patient counseling strategies, and ultimately reducing the burden of morbidity associated with DKA in the region. Therefore, we aimed to assess awareness of DKA symptoms, management, and diabetes-related complications, in addition to adherence, among patients with type 1 diabetes attending outpatient clinics at KFSH.

## Materials and methods

Study design, setting, and sampling

This cross-sectional study was conducted at a single tertiary care center using convenience sampling to evaluate awareness of DKA symptoms, management, prevention, and diabetes-related complications, as well as adherence, among patients among adult patients with type 1 diabetes mellitus attending outpatient clinics at KFSH. Data were collected from January 2025 to December 2025 using a structured questionnaire conducted via telephone interviews after obtaining participants’ consent.

Inclusion and exclusion criteria

Adult patients diagnosed with type 1 diabetes mellitus and aged ≥ 18 years were eligible for inclusion. Patients were excluded if they had cognitive impairment (e.g., dementia or other mental disabilities) that could compromise the reliability of questionnaire responses or had incomplete hospital records.

Data collection instrument 

Data were collected using a structured questionnaire. The questionnaire was interviewer-administered through telephone interviews conducted by the study investigators. All questions were read to participants as written in the questionnaire, and responses were recorded directly by the interviewer using a standardized approach. The questionnaire gathered sociodemographic data, including age, sex, marital status, educational level, and type of treatment. It also included 11 structured items assessing awareness of DKA-related symptoms, including common manifestations such as fatigue, polyuria, polydipsia, nausea, vomiting, abdominal pain, blurred vision, fruity breath odor, palpitations, rapid breathing, and confusion or loss of consciousness.

Awareness of DKA management and prevention was assessed using 11 items that addressed the definition of DKA, its emergency nature, the importance of glycemic control, appropriate actions when DKA is suspected, and common precipitating factors, including insulin omission, dietary errors, infection, inadequate glucose monitoring, and dehydration. Awareness of diabetes-related complications was assessed using 10 items, including microvascular and macrovascular complications such as visual impairment, chronic kidney disease, neuropathy, limb ulcers and amputations, cardiovascular disease, stroke, cognitive decline, skin infections, gastroparesis, and erectile dysfunction. Adherence to recommended diabetes self-care practices was assessed using 10 items evaluating insulin administration, blood glucose monitoring, dietary compliance, and regular follow-up practices. 

The questionnaire was developed by adapting items from previously published studies [8,13]. Content validity was established through expert review by specialists, who evaluated item relevance, clarity, and cultural appropriateness. The instrument was then pilot-tested on 20 patients with type 1 diabetes mellitus. Feedback from the pilot phase led to minor refinements in wording and response options to improve clarity and reduce ambiguity. Data from the pilot test were not included in the final analysis. Each item had a single correct response, which was coded as 1, whereas incorrect or undesirable responses were coded as 0. The total domain scores were calculated by summing the item scores within each domain, with higher scores indicating greater awareness or adherence. Patients’ awareness and adherence levels were classified using the 50% and 75% cutoff points, as specified by Eltom et al. [13]. Scores < 50% of the maximum possible score were considered poor; scores between 50% and 75% were considered moderate; and scores > 75% were considered indicative of good awareness or adherence.

Ethical approval

Approval was obtained from the hospital administration and the Regional Research Ethics Committee of Qassim Province (approval number: H-04-Q-001). 

Statistical analysis

Descriptive statistics were generated using SPSS Statistics version 26 (IBM Corp., Armonk, NY) software. Categorical variables are summarized as frequencies and percentages, whereas continuous variables are presented as mean ± standard deviation (SD). The normality of all awareness and adherence scores was assessed using a Kolmogorov-Smirnov test, which confirmed a non-normal distribution of all composite scores. Therefore, non-parametric statistical methods were used in this study. Comparisons between two independent groups were performed using a Mann-Whitney U test, whereas comparisons across more than two groups were conducted using a Kruskal-Wallis H test. Associations between the awareness domains and diabetes adherence were examined using Spearman's rank correlation coefficients. Statistical significance was set at p < 0.05, and a p-value < 0.01 was considered highly statistically significant.

## Results

We enrolled 206 patients with type 1 diabetes. Table 1 presents the sociodemographic characteristics of the patients. The study cohort was predominantly young, with 104 (50.5%) patients aged 21-25 years and 58 (28.2%) aged 18-20 years. Men constituted the majority of the participants (114, 55.3%). Most patients were single (170, 82.5%). Regarding education, 118 (57.2%) had a secondary education or lower level of education, and 85 (41.3%) had a bachelor's degree. Regarding treatment modality, 179 (86.9%) used multiple daily insulin injections, while 27 (13.1%) used an insulin pump.

**Table 1:** Sociodemographic characteristics of the patients with type 1 diabetes (n = 206)

Study variables	N (%)
Age group, years	
18–20	58 (28.2%)
21–25	104 (50.5%)
≥ 26	44 (21.4%)
Sex	
Male	114 (55.3%)
Female	92 (44.7%)
Marital status	
Single	170 (82.5%)
Married	36 (17.5%)
Educational level	
Secondary or below	118 (57.3%)
Bachelor's degree	85 (41.3%)
Postgraduate	3 (1.5%)
Type of treatment	
Insulin pump	27 (13.1%)
Multiple insulin injections	179 (86.9%)

As shown in Table 2, awareness of the common symptoms of DKA was generally high for early nonspecific manifestations, with 186 (90.3%) identifying fatigue, 185 (89.8%) identifying frequent urination, 181 (87.9%) identifying extreme thirst, and 179 (86.9%) identifying nausea as symptoms. Awareness rates declined for more specific or severe indicators, particularly blurred vision (79, 38.3%), fruity breath odor (72, 35%), palpitations (67, 32.5%), rapid breathing (61, 29.6%), and confusion or loss of consciousness (41, 19.9%). The mean symptom awareness score was 6.70 ± 2.05, with most patients demonstrating moderate awareness (130, 63.1%). Awareness of DKA management and prevention was stronger, with all patients (206, 100%) correctly identifying DKA as a state of high blood sugar and acidosis, and high proportions recognizing the importance of HbA1c control (191, 92.7%), the emergency nature of DKA (183, 88.8%), and the need for urgent medical attention (176, 85.4%). Key precipitating factors were also well recognized, particularly insulin omission (191, 92.7%), dietary errors (143, 69.4%), and infection (120, 58.3%). However, fewer patients identified precipitating factors, including inadequate glucose monitoring (90, 43.7%), dehydration (65, 31.6%), the menstrual cycle (21, 10.2%), and physical exertion (20, 9.7%).

**Table 2:** Assessment of awareness of DKA-related symptoms, management, prevention, and diabetes-related complications (n = 206) DKA: diabetic ketoacidosis; SD: standard deviation; HbA1c: glycated hemoglobin

Variables	Values
Awareness of DKA-related symptoms, n (%)	
Feeling tired/fatigue (yes)	186 (90.3%)
Frequent urination (yes)	185 (89.8%)
Extreme thirst (yes)	181 (87.9%)
Nausea (yes)	179 (86.9%)
Vomiting (yes)	167 (81.1%)
Abdominal pain (yes)	163 (79.1%)
Blurred vision (yes)	79 (38.3%)
Distinctive breath smell (AKA "fruity breath") (yes)	72 (35.0%)
Palpitations (yes)	67 (32.5%)
Difficulty breathing or rapid breathing (yes)	61 (29.6%)
Confusion or loss of consciousness (yes)	41 (19.9%)
Awareness of DKA-related symptom score (mean ± SD)	6.70 ± 2.05
Level of awareness about symptoms of DKA, n (%)	
Poor	35 (17.0%)
Moderate	130 (63.1%)
Good	41 (19.9%)
Awareness of DKA management and prevention, n (%)	
1. The statement "DKA is a state of high blood sugar and acidity in the blood" is true (yes)	206 (100%)
2. The statement "Maintaining the HbA1c within the target reduces the incidence of DKA" is true (yes)	191 (92.7%)
3. How would you describe DKA? (an emergency complication of diabetes that requires urgent treatment)	183 (88.8%)
4. What should you do if you suspect you have DKA? (Go to the hospital or call emergency services immediately)	176 (85.4%)
What factors do you think can contribute to DKA? n (%)	
5. Forgetting to take insulin injections (yes)	191 (92.7%)
6. Dietary errors (i.e., binge eating, not following a diabetic diet) (yes)	143 (69.4%)
7. Infection (bacterial, viral, or fungal) (yes)	120 (58.3%)
8. Not checking blood sugar levels regularly (yes)	90 (43.7%)
9. Dehydration (yes)	65 (31.6%)
10. Menstrual cycle (yes)	21 (10.2%)
11. Physical exertion (yes)	20 (9.7%)
Total awareness of DKA management and prevention score (mean ± SD)	6.83 ± 1.47
Level of awareness about DKA management and prevention, n (%)	
Poor	34 (16.5%)
Moderate	148 (71.8%)
Good	24 (11.7%)
Awareness of diabetes-related complications, n (%)	
Decreased vision (yes)	196 (95.1%)
Chronic kidney disease and need for dialysis (yes)	182 (88.3%)
Chronic numbness of limbs (yes)	176 (85.4%)
Ulcers and amputations of limbs (yes)	108 (52.4%)
Heart disease (yes)	81 (39.3%)
Stroke (yes)	74 (35.9%)
Cognitive decline (yes)	69 (33.5%)
Skin infections (yes)	54 (26.2%)
Slow stomach emptying (yes)	47 (22.8%)
Erectile dysfunction (yes)	43 (20.9%)
Total awareness of diabetes-related complications score (mean ± SD)	5.00 ± 1.93
Level of awareness about diabetes-related complications, n (%)	
Poor	145 (70.4%)
Moderate	46 (22.3%)
Good	15 (7.3%)

**Figure 1:**
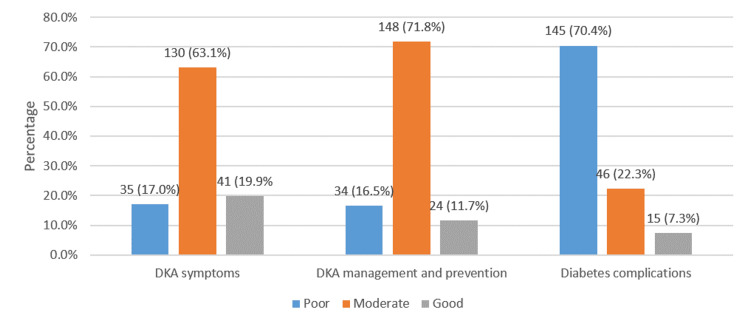
Level of awareness of DKA symptoms, management, prevention, and complications DKA: diabetic ketoacidosis

The mean management awareness score was 6.83 ± 1.47, with most patients demonstrating moderate awareness (148, 71.8%). By contrast, awareness of diabetes-related complications was highest for decreased vision (196, 95.1%), kidney disease (182, 88.3%), and chronic limb numbness (176, 85.4%), but substantially lower for skin infections (54, 26.2%), gastroparesis (47, 22.8%), and erectile dysfunction (43, 20.9%). The mean complication awareness score was 5.00 ± 1.93, with most patients classified as having poor awareness (145, 70.4%). The overall levels of awareness regarding DKA symptoms, management and prevention, and complications are presented in Figure 1.

**Figure 2:**
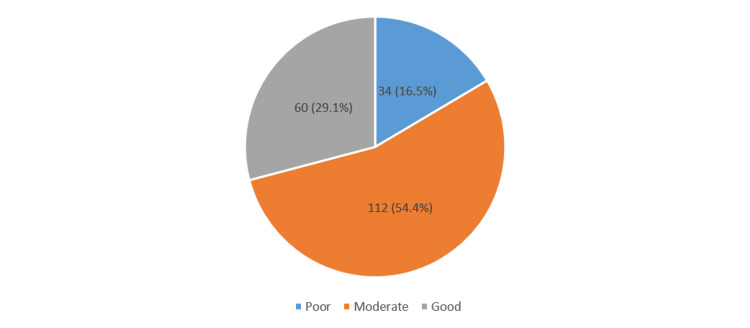
Level of adherence

As shown in Table 3, overall adherence among patients with type 1 diabetes was mixed, with strong adherence to some clinical practices but notable gaps in lifestyle-related behaviors. Most patients (139, 67.5%) reported maintaining an HbA1c level ≤ 8%. Routine monitoring practices were generally well maintained: 174 (84.5%) underwent HbA1c testing every three to six months, 163 (79.1%) visited their diabetes physician at recommended intervals, and 157 (76.2%) attended annual ophthalmology evaluations. However, only 106 (51.5%) checked their random blood sugar level at least four times daily, and 57 (27.7%) reported missing their medications more than once per week. Lifestyle adherence was considerably lower, with only 63 (30.6%) following a diabetes-specific diet and 86 (41.7%) engaging in regular exercise. The mean adherence score was 6.25 ± 1.78, with 112 (54.4%), 60 (29.1%), and 34 (16.5%) patients demonstrating moderate, good, and poor adherence, respectively (Figure 2).

**Table 3:** Assessment of adherence (n = 206) DKA: diabetic ketoacidosis; HbA1c: glycated hemoglobin; SD: standard deviation

Adherence items	Values
1. How often do you do an HbA1c level test? (every 3–6 months) n (%)	174 (84.5%)
2. Have you had DKA episodes more than once last year? (no) n (%)	174 (84.5%)
3. How often do you visit your diabetes physician? (every 3–6 months) n (%)	163 (79.1%)
4. How often do you visit your ophthalmologist? (annually) n (%)	157 (76.2%)
5. Do you frequently miss taking your medication >1 time per week? (no) n (%)	149 (72.3%)
6. What is your last HbA1c level? (≤ 8%) n (%)	139 (67.5%)
7. How often do you check for blood glucose? (≥4 times/day) n (%)	106 (51.5%)
8. Do you do regular exercise (150 min/week)? (yes) n (%)	86 (41.7%)
9. Do you follow sick day protocol when you are sick? (yes) n (%)	75 (36.4%)
10. Do you follow a diabetic diet? (yes) n (%)	63 (30.6%)
Total adherence score (mean ± SD)	6.25 ± 1.78
Level of adherence, n (%)	
Poor	34 (16.5%)
Moderate	112 (54.4%)
Good	60 (29.1%)

As shown in Table 4, all assessed domains of DKA awareness demonstrated significant positive intercorrelations and were also positively correlated with adherence (p < 0.01). Awareness of DKA symptoms showed a moderate correlation with awareness of DKA management and prevention (Rs = 0.441), awareness of diabetes-related complications (Rs = 0.393), and adherence (Rs = 0.430), indicating that patients who recognized early manifestations of DKA were more knowledgeable about its management, long-term risks, and adherence. Moderate correlations were found between adherence and awareness of DKA-related symptoms (Rs = 0.430) and awareness of management and prevention (Rs = 0.333), indicating that more informed patients were more likely to adhere to recommended diabetes care practices. The association between adherence and awareness of diabetes-related complications was also positive and statistically significant (Rs = 0.194), indicating a small but significant association between knowledge of long-term risks and the adoption of appropriate self-management practices.

**Table 4:** Spearman's correlation coefficient between awareness of DKA-related symptoms, management, prevention, complications, and adherence (n = 206) ^*^Correlation is significant at the 0.01 level (two-tailed) DKA: diabetic ketoacidosis

Variables	Awareness			Diabetes adherence, Rs value
	Symptoms, Rs value	Management and prevention, Rs value	Diabetes-related complications, Rs value	
Awareness of symptoms of DKA	1			
Awareness of DKA management and prevention	0.441^*^	1		
Awareness of diabetes-related complications	0.393^*^	0.360^*^	1	
Adherence	0.430^*^	0.333^*^	0.194^*^	1

As shown in Table 5, most sociodemographic factors were not significantly associated with awareness of DKA symptoms, management, and prevention, or diabetes-related complications. Awareness of DKA symptoms did not differ significantly according to age (p = 0.242), marital status (p = 0.096), or educational level (p = 0.226). However, women demonstrated significantly higher DKA symptom awareness scores than men (7.08 ± 2.05 vs. 6.40 ± 2.01, respectively; p = 0.046). Patients using an insulin pump showed significantly higher DKA symptom awareness scores than those using multiple daily insulin injections (8.33 ± 2.35 vs. 6.46 ± 1.89, respectively; p < 0.001), as did those with an HbA1c value ≤ 8% compared with those with an HbA1c value > 8% (6.99 ± 1.87 vs. 6.06 ± 2.29, respectively; p = 0.008). Awareness of DKA management and prevention was generally similar across age groups (p = 0.279), sex (p = 0.110), marital status (p = 0.382), and education levels (p = 0.220).

**Table 5:** Association between sociodemographic characteristics and awareness of DKA symptoms, management, prevention, and complications among patients with type 1 diabetes (n = 206) ^§^P-value was calculated using the Kruskal-Wallis H-test. ^‡^P-value was calculated using the Mann-Whitney Z-test. ^*^Significant at p < 0.05 level DKA: diabetic ketoacidosis; SD: standard deviation; DM: diabetes mellitus; HbA1c: glycated hemoglobin

Factor	Awareness		
	Symptoms score (11), mean ± SD	Management and prevention score (11), mean ± SD	DM complications score (10), mean ± SD
Age group, years			
18–20	6.86 ± 1.68	6.72 ± 1.53	4.53 ± 2.01
21–25	6.55 ± 1.98	7.02 ± 1.26	4.98 ± 1.75
≥ 26	6.86 ± 2.60	6.50 ± 1.79	5.66 ± 2.08
H-test; p-value^§^	H = 2.835; p = 0.242	H = 2.556; p = 0.279	H = 9.450; p = 0.009^*^
Sex			
Men	6.40 ± 2.01	6.68 ± 1.36	5.19 ± 2.08
Women	7.08 ± 2.05	7.00 ± 1.59	4.76 ± 1.71
Z-test; p-value^‡^	Z = 1.998; p = 0.046^*^	Z = 1.600; p = 0.110	Z = 1.461; p = 0.144
Marital status			
Single	6.81 ± 2.06	6.85 ± 1.48	4.97 ± 1.94
Married	6.22 ± 1.99	6.69 ± 1.47	5.14 ± 1.91
Z-test; p-value^‡^	Z = 1.663; p = 0.096	Z = 0.874; p = 0.382	Z = 0.554; p = 0.580
Educational level			
Secondary or below	6.50 ± 1.89	6.69 ± 1.43	4.74 ± 1.68
Bachelor's or higher	6.98 ± 2.23	7.00 ± 1.52	5.35 ± 2.18
Z-test; p-value^‡^	Z = 1.211; p = 0.226	Z = 1.228; p = 0.220	Z = 2.357; p = 0.018^*^
Type of treatment			
Insulin pump	8.33 ± 2.35	7.37 ± 1.92	6.74 ± 1.38
Multiple insulin injections	6.46 ± 1.89	6.74 ± 1.38	6.48 ± 2.29
Z-test; p-value^‡^	Z = 3.613; p < 0.001^*^	Z = 2.270; p < 0.001^*^	Z = 3.610; p < 0.001^*^
Last HbA1c			
≤ 8%	6.99 ± 1.87	6.99 ± 1.26	5.01 ± 1.65
> 8%	6.06 ± 2.29	6.45 ± 1.83	4.97 ± 2.46
Z-test; p-value^‡^	Z = 2.669; p = 0.008^*^	Z = 2.288; p = 0.022^*^	Z = 0.240; P = 0.810

Patients with an HbA1c level ≤ 8% compared with those with an HbA1c level > 8% (6.99 ± 1.26 vs. 6.45 ± 1.83, respectively; p = 0.022), and those using an insulin pump compared with those using multiple daily insulin injections (7.37 ± 1.92 vs. 6.74 ± 1.38, respectively; p < 0.001), showed significantly higher awareness of DKA management and prevention. Awareness of diabetes-related complications was significantly associated with age (p = 0.009), with patients aged 26 years or older demonstrating higher scores (5.66 ± 2.08). Awareness of diabetes-related complications was significantly higher among patients with higher levels of education than among those with lower levels of education (5.35 ± 2.18 vs. 4.74 ± 1.68, respectively; p = 0.018) and among those using an insulin pump compared with those using multiple daily insulin injections (6.48 ± 2.29 vs. 4.74 ± 1.38, respectively; p < 0.001). No significant differences were observed according to sex (p = 0.144), marital status (p = 0.580), or HbA1c level (p = 0.810) with respect to awareness of diabetes-related complications.

As shown in Table 6, adherence varied significantly across sociodemographic characteristics. Women had significantly higher adherence (6.77 ± 1.73) than men (5.83 ± 1.72; p < 0.001). Adherence was not significantly associated with age (p = 0.187), marital status (p = 0.229), or educational level (p = 0.677). However, adherence was significantly higher among patients using an insulin pump compared with those using multiple daily insulin injections (7.00 ± 1.94 vs. 6.14 ± 1.73, respectively; p = 0.021). Additionally, adherence was significantly associated with the most recent HbA1c level (p < 0.001), with patients having an HbA1c level ≤ 8% demonstrating higher adherence (6.89 ± 1.38) than those with an HbA1c level > 8% (4.84 ± 1.77), indicating better adherence among patients with more optimal glycemic control.

**Table 6:** Association between adherence and patients’ sociodemographic characteristics (n = 206) ^§^P-values were calculated using a Mann-Whitney Z-test. ^*^Significant at p < 0.05 SD: standard deviation; HbA1c: glycated hemoglobin

Factor	Adherence score (10), mean ± SD	H/Z test	P-value^§^
Age group, years			
18–20	6.02 ± 1.54	H = 3.351	0.187
21–25	6.47 ± 1.68		
≥ 26	6.05 ± 2.24		
Sex			
Men	5.83 ± 1.72	Z = 3.955	<0.001^*^
Women	6.77 ± 1.73		
Marital status			
Single	6.32 ± 1.76	Z = 1.202	0.229
Married	5.89 ± 1.85		
Educational level			
Secondary or below	6.25 ± 1.82	Z = 0.417	0.677
Bachelor's or higher	6.26 ± 1.74		
Type of treatment			
Insulin pump	7.00 ± 1.94	Z = 2.310	0.021^*^
Multiple insulin injections	6.14 ± 1.73		
Last HbA1c			
≤ 8%	6.89 ± 1.38	Z = 7.385	<0.001^*^
> 8%	4.84 ± 1.77		

## Discussion

Most patients used multiple daily insulin injections rather than insulin pumps, which is consistent with Saudi DKA awareness studies reporting that multiple daily injections remain the predominant insulin regimen among surveyed patients [7,11]. Similar demographic patterns have been observed in Sudan, where DKA-related clinic attendance is more frequent among younger adults [14]. Awareness of early nonspecific symptoms such as fatigue, polyuria, polydipsia, and nausea was relatively high. This is in line with the findings from Germany, where Hepprich et al. reported that persons with type 1 diabetes had significant awareness of common DKA symptoms [6]. Conversely, awareness of more specific or severe symptoms, such as fruity breath, rapid breathing, and altered consciousness, was low, reflecting gaps reported in Riyadh and Makkah [7,8].

A similar limited awareness of late-stage DKA symptoms has also been reported in studies from Sudan [14]. Clinical guidelines highlight the need for structured education on early and late symptoms [1,2] and emphasize that DKA is a complex metabolic acidosis rather than simply a hyperglycemic state alone. Our cohort had an average overall symptom awareness score, which is consistent with findings in Khartoum [14]. A study conducted in Khobar also identified gaps in DKA symptom awareness, but it examined caregivers of pediatric patients rather than adults [9], suggesting that educational gaps may exist across different populations.

The majority of participants correctly identified insulin omission, dietary errors, and infection as precipitating factors. This is consistent with a study conducted in Riyadh, where insulin omission was commonly recognized as a major trigger [7]. Studies of recurrent DKA have shown that even though patients often remember receiving education, they may still struggle with compliance due to psychosocial barriers [4]. Limited knowledge about dehydration and glucose monitoring was reported in both Riyadh and Sudan [7,14]. Eltom et al. also found poor awareness of secondary risk factors such as dehydration and poor glucose monitoring despite good knowledge of insulin omission [13]. Recent reviews have suggested that DKA management challenges persist for some patient populations, such as those with comorbidities or limited access to specialized care [15]. These patterns suggest that patient education may disproportionately emphasize insulin omission while underrepresenting other clinically relevant triggers.

Awareness of diabetes-related complications was substantially lower than awareness of DKA symptoms or management. Microvascular complications such as retinopathy, nephropathy, and neuropathy were more frequently recognized, whereas awareness of autonomic and dermatologic complications, including gastroparesis, skin infections, and erectile dysfunction, was limited. Although studies from Khobar and Sudan have reported gaps in diabetes-related knowledge [9,14], these studies mainly assessed DKA-specific awareness rather than detailed complication profiles. However, the low recognition of autonomic complications in our cohort is consistent with global evidence that autonomic complications are widely underdiagnosed and often underemphasized in patient and clinician education [16]. The low mean awareness score regarding complications indicates the need for more comprehensive education covering the full spectrum of diabetes-related complications.

Adherence patterns in our cohort were mixed, with good adherence to structured clinical follow-up, including HbA1c testing, physician visits, and ophthalmology evaluations, but poorer adherence to lifestyle-related behaviors. Similar findings have been reported in Saudi studies, where adherence to scheduled clinical monitoring was generally better than adherence to diet, exercise, and sick-day rules [7,11,12]. Lifestyle behaviors are consistently identified as the most challenging components of diabetes self-management worldwide [5]. Studies from Sudan also describe limited adherence to lifestyle interventions despite adequate clinical follow-up [14]. The moderate overall adherence score in our cohort is consistent with findings from Riyadh and Makkah, where adherence was influenced by behavioral and psychosocial factors [7,8]. The association of higher adherence with optimal HbA1c levels is consistent with evidence that self-care behaviors contribute to improved glycemic control [5].

The positive correlation between awareness domains and adherence further supports the well-established relationship between knowledge and self-management behaviors. Studies from Germany and Sudan have shown that increased awareness is associated with improved compliance and reduced DKA events [6,14]. Recent literature suggests that structured, repeated educational interventions can help improve awareness and potentially reduce the likelihood of DKA recurrence [13,16]. Research targeting physicians also underscores the importance of standardized DKA management protocols, suggesting that uniform provider education may indirectly translate into better patient outcomes by ensuring consistent counseling practices [17].

Women had significantly greater awareness of DKA symptoms and higher adherence than men, consistent with Saudi studies reporting gender-related differences in DKA awareness and diabetes self-care behaviors [7,11,12]. Users of insulin pumps had better awareness and adherence, which may be explained by the more comprehensive diabetes education usually provided to individuals using advanced insulin-delivery methods. The International Society for Pediatric and Adolescent Diabetes (ISPAD) guidelines primarily address the pediatric population but do mention the educational needs associated with pump therapy [2], which could be conceptually relevant for adult users. Older age was associated with increased awareness of diabetes-related complications, a trend also observed in Sudan [14]. Although some Saudi studies have reported higher awareness among university-educated patients [7,12], our cohort did not show consistent associations between education level and awareness. Recent reviews suggest that education level alone may not predict awareness unless accompanied by structured diabetes education programs [16].

Overall, the findings indicated moderate awareness of DKA symptoms, management, and prevention, but poor awareness of DKA-related complications. Furthermore, patients demonstrated only moderate treatment adherence. These gaps align with regional and international trends and suggest that current educational approaches may not be sufficiently targeted or patient-centered. Enhancing structured diabetes education, especially regarding early and late symptoms of DKA, diabetes-related complications, and behavioral barriers to lifestyle adherence, may decrease the burden of DKA and improve long-term outcomes. Alshareef et al. emphasized the importance of caregiver awareness in recognizing DKA symptoms early in pediatric populations [18]. Although their results apply to caregivers of children and adolescents, they suggest that family-centered educational models could also have positive effects on adult patients by improving support systems and possibly reducing DKA recurrence. Incorporating these approaches into the care of adults with diabetes could improve patient engagement and outcomes.

Study limitations 

This study has several limitations that should be considered when interpreting the findings. First, the cross-sectional design limits causal inference, making it impossible to determine whether higher awareness leads to better adherence or whether better adherence contributes to higher awareness. Second, this was a single-center study, which may limit the generalizability of the findings to other populations and healthcare settings. Third, the use of convenience sampling may have introduced selection bias, as participants who were available and willing to participate may differ from the broader population of patients with type 1 diabetes.

Furthermore, the use of self-reported data may have introduced recall and social desirability biases, potentially affecting the accuracy of responses. Although the questionnaire underwent expert-based content validation and pilot testing before implementation, additional psychometric evaluation, including assessments of internal consistency and construct validity, may further strengthen the instrument in future studies. Lastly, although sociodemographic factors associated with adherence were assessed, other behavioral and psychosocial determinants that may influence diabetes self-management were not evaluated in this study and should be explored in future research.

## Conclusions

This study identified substantial gaps in patient awareness of severe DKA symptoms, precipitating factors, and diabetes-related complications among individuals with type 1 diabetes. Although routine clinical follow-up practices, such as HbA1c monitoring, physician visits, and ophthalmologic screening, were generally well maintained, lifestyle-related adherence, including diet, exercise, and sick-day management, remained limited. Significant associations observed in this study further underscore the importance of targeted education: women, insulin pump users, and patients achieving an HbA1c level ≤ 8% consistently demonstrated higher awareness and stronger adherence, while older and better-educated patients showed greater recognition of diabetes complications. The strong positive correlations between awareness domains and adherence highlight the association between patient knowledge and self-management behaviors, suggesting that improving awareness may be an important component of diabetes care. Addressing these gaps through structured, repeated, and culturally tailored diabetes education programs may enhance self-management, reduce acute metabolic crises, and ultimately improve the quality of life for patients with type 1 diabetes.
